# An Analysis of Foams Produced from Recycled Polyolefins and Low-Cost Foaming Agents: Benchmarking Using Pore Size, Distribution, Shear Effects, and Thermal Properties

**DOI:** 10.3390/polym17091270

**Published:** 2025-05-06

**Authors:** Krishnamurthy Prasad, Fareed Tamaddoni Jahromi, Shammi Sultana Nisha, John Stehle, Emad Gad, Mostafa Nikzad

**Affiliations:** 1 School of Engineering, Swinburne University of Technology, Hawthorn, VIC 3122, Australia; krishnamurthyprasad@swin.edu.au (K.P.); ftamaddonijahromi@swin.edu.au (F.T.J.); snisha@swin.edu.au (S.S.N.); egad@swin.edu.au (E.G.); 2 Robovoid Pty Ltd., Carnegie, VIC 3163, Australia; johnstehle@robovoid.com

**Keywords:** recycled polyolefins, foaming, pore size distribution, density, shear

## Abstract

Foamed specimens were fabricated from virgin and recycled polyethylenes (linear low-density polyethylene, LLDPE, and low-density polyethylene, LDPE) using low-cost citric acid and sodium bicarbonate foaming agents. The foaming agents chosen showed decomposition behaviour either without phase change (sodium bicarbonate, NaB) or liquefaction followed by decomposition (citric acid, CA). The manufactured polyethylene foams were then benchmarked against a polyurethane foam. Two types of mixing were used prior to foaming, viz., solid-state pulverisation or high-shear internal mixing, and the effect of mixing on properties critical for foam viability were analysed. These properties included density, pore size, shape and distribution, crystallinity, and porosity. It was found that the virgin LLDPE and recycled LDPE showed similar trends in terms of narrow pore size distribution and reduced crystallinity, while the recycled LLDPE tended towards more pore coalescence. This difference in behaviour was attributed to the more mixed phase nature of the recycled LLDPE as opposed to the majorly single-phase virgin LLDPE and recycled LDPE. Lowered densities obtained for the NaB foaming compared to CA can be speculated to be because of the ionic and simple NaB decomposition as opposed to the complex radical pathway for the CA decomposition.

## 1. Introduction

**Figure 1:**
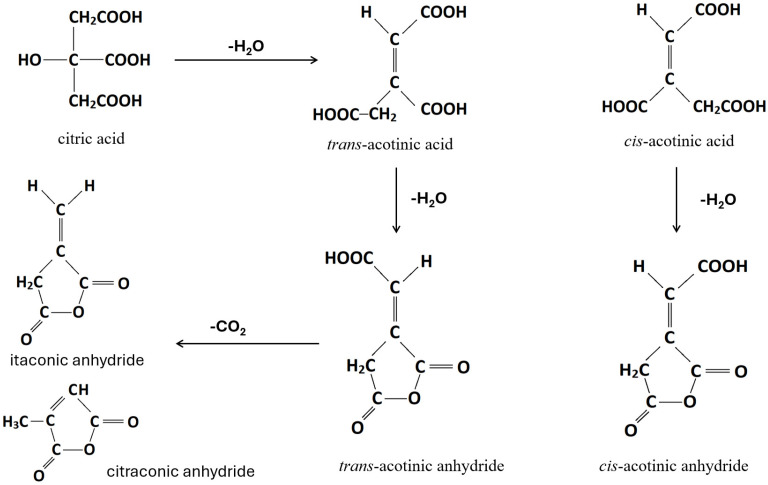
Scheme of degradation reactions and products thereof of citric acid.

The structure, morphology, and properties of foam depend on a number of factors, including the rheological and thermodynamic properties of the polymer/gas system, the gas content, the processing conditions, and the foaming dynamics. It is crucial to control the rheological behaviour of macromolecular viscoelastic materials that contain dissolved gases at high concentrations at thermodynamic conditions conducive to the formation of gas bubbles within the melt in order to optimise manufacturing processes of cellular polymers [1,2,3]. The final morphology of foam is determined by the nucleation and growth rate of the polymeric melt, which are in turn influenced by its physical and rheological properties. There are several variables involved in the polymer/gas system, such as surface tension, elongational viscosity, solubility, and diffusivity of gas [4]. It is expected that the enhancement of the viscoelastic properties will inhibit cell growth and reduce cell wall ruptures and collapses. In regard to polymers, foamability is strongly influenced by their rheological characteristics [4,5]. In order to achieve successful foaming, certain characteristics are commonly reported as being necessary, including high melt strength to with-stand elongational stresses during the growth of bubbles [6,7] It is also important to consider the size of the processing window, which is determined by the difference between the melting temperature (T_m_) and the crystallisation temperature (T_c_) in the case of semicrystalline polymers or the glass transition temperature (T_g_) in the case of amorphous polymers [3,8]. The most widely used polymeric materials to fabricate foams are polyurethane (PU) [9], followed by LDPE + EVA (ethylene-vinyl acetate) blends [9], polyvinyl chloride (PVC) [10], EVA [10], and polyester + PE blends [10]. Apart from PU, the development of foam materials based on polyolefins and other polymers require the utilisation of so-called chemical blowing agents (CBA) that are based on either exothermic mechanism (azodicarbonamide) or endothermic mechanism (alkali carbonates or citric acid esters). A well-known phenomenon is that dissolving the blowing agent can result in a plasticising effect on polymer matrixes [3,8], which leads to a decrease in T_g_ and T_m_. The gas dissolved in the melt can cause a rapid increase in viscosity during bubble growth if the processing temperature is near the glass transition temperature of an amorphous polymer [11,12]. The situation is more complex in semicrystalline polymers, since the increase in free volume affects both the crystallisation temperature and its kinetics [11,12]. By preventing cell coalescence, crystallisation can also improve the foaming of low melt strength polymers. For the foam to have a high expansion ratio and a considerable cell density, it is necessary to maintain an optimal crystallisation degree during processing. These may be manufactured through either extrusion or injection moulding techniques [13,14]. There has been a push for recognising the foaming abilities of lower cost foaming agents such as sodium bicarbonate (NaB) and citric acid (CA), and their respective decomposition/foaming mechanisms are shown in Equation (1) and Figure 1, respectively [15,16].

(1) $$2NaHCO_{3}\to {Na}_{2}CO_{3}+H_{2}O+CO_{2}$$

To this end, Ruiz et al. [17] manufactured foams of PP with these CBAs in pellet form. A concentration range of 1–4% by weight was used with a temperature profile of 160–210 °C in injection moulding, as the activation temperature of the blowing agents lies within that range. The densities of the formed foams were all in the range of 0.87 g/cm^3^ compared to the raw material density of 0.91 g/cm^3^.

A foam of PP (linear copolymer, branched homopolymer) using a mixed CBA (comprising of 56/44 mixture of NaHCO_3_ and citric acid) was fabricated by Fasihi et al. [18]. The PP and the CBAs were premixed using a high-speed dry mixer followed by extrusion on a single screw extruder. The zone temperature profiles of the extruder, starting from the feeding zone to the die were set to be 140 °C, 170 °C, 190 °C, 210 °C, and 170 °C, respectively. Foam densities varied from 0.15 to 0.47 g/cm^3^, with cell thicknesses and cell density also being able to be tuned with a high degree of control.

Li et al. [19] fabricated foams of high density polyethylene (HDPE) using a range of exothermic and endothermic CBAs using a single-screw extruder (length/diameter ratio = 30/1, C.W. Brabender). Average cell sizes of the foam fabricated in the report by Li et al. [19] ranged from 50 to 1300 microns, indicating the wide range of foam properties possible from the foaming of a polyolefin. Core back injection moulding with injection temperature set at 230 °C using 2% by weight of CBA was used by Ruiz et al. [20] to manufacture foams of PP. A total of 0.02 g of CBA was introduced per g of PP, which gave a relative value of gas of 0.006 g_gas_/g_PP_ for CBA-1 and 0.007 g_gas_/g_PP_ for CBA-2. Similar to Rui et al. [17], CBA-1 was 35 wt% of citric acid and 35 wt% of NaHCO_3_, 30% PE, while CBA-2 was 70% citric acid, 30% PE. Part of the gas generated corresponded to H_2_O vapor that after foaming and cooling remains in the samples as condensed water vapor. Specifically, about 30% of all the gas generated corresponded to H_2_O vapor and the rest was CO_2_. The maximum quantity of CO_2_ that can be diluted at the temperature and pressure conditions in the nozzle was about 0.05 g_CO2_/g_PP._ However, no analysis of mechanical properties or density characteristics of the foam was demonstrated.

In terms of recycled polymers, Greco et al. [21] used recycled gypsum and a commercial foaming agent (Ferrocell AZC13R) as the foaming agent for recycled HDPE and film-grade recycled LDPE using extrusion. The use of recycled gypsum offered a unique foaming pathway shown in Equations (2) and (3).

(2) $$CaSO_{4}\cdot 2H_{2}O\to CaSO_{4}\cdot 0.5H_{2}O+1.5H_{2}O$$

(3) $$CaSO_{4}\cdot 0.5H_{2}O\to CaSO_{4}+0.5H_{2}O$$

It has to be noted that the density trends studied in Greco et al. [21] have to be juxtaposed with stress–strain behaviour to understand cushioning effects, but no such analysis was conducted. The results obtained by Greco et al. [21], viz., optimisation of experimental design, indicated the following:

1. Temperatures in the extruder were found to have a weak influence on the density of extruded products.
2. There was a strong dependence of the density of extrudates on the die temperature.
3. In addition, the density of extrudates increased with screw speed.
4. Foam/extrudate density decreased with die diameter or length if the ratio L/D was held constant.
5. Density and foaming efficiencies (based on the quantity of unreacted CaSO_4_) both showed a decreasing trend with an increase in gypsum quantity in recycled HDPE.
6. Adding recycled LDPE at various concentrations to recycled HDPE had a major influence on extrudate density, indicating a significant effect of comingled recycled raw material nature on the resultant foam properties.

Based on the limited available literature, it can therefore be seen that the majority of work conducted on polyolefin foam fabrication involves using CBAs and virgin polymers; comparable work on recycled polyolefins is low, barring those of Greco and Maffezeli [21]. In addition, the simultaneous use of real-life post-consumer recycled polyolefins as a raw material and benchmarking with a virgin polymer under the same conditions is lacking. Therefore, in this work, these gaps were addressed by fabricating foams of post-consumer polyethylenes (LLDPE and LDPE) and comparing them with a foam fabricated using virgin LLDPE and the CBAs used were low-cost food grade sodium bicarbonate and citric acid to further add on to the sustainability of this work. Analysis was conducted of the pore sizes, distribution, density, and thermal properties and contrasted with those seen for commercial foams based on PE and PU to gain an understanding of the influence of these individual parameters on the foam behaviour. Finally, the effect of shear on the foams was studied by performing the fabrication under two separate environments, viz., low shear pulverisation and dry mixing, as well as high shear internal mixing.

## 2. Materials and Methods

**Figure 2:**
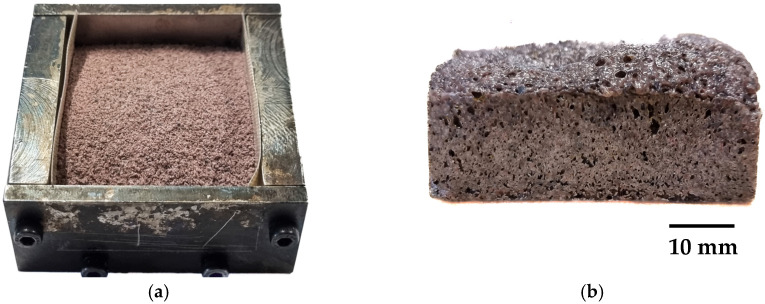
Foaming of recycled LDPE with citric acid. (**a**) Dry mixed polymer and foaming agent powder before being placed in the oven. (**b**) Foam product after being removed from the oven and the mould.

Recycled LDPE and LLDPE were obtained from GT Recycling Pty Ltd., Geelong, Victoria, while rotomolding-grade virgin LLDPE (V_LLDPE) was obtained from Qenos Ltd., Pakenham, Victoria, Australia. The MFIs of the received recycled LDPE and LLDPE were 1.84 g/10 min and 11.89 g/10 min, respectively. The MFI was measured at 190 °C using a weight of 2.16 Kg on a Ceast modular line melt flow machine as per ASTM D1238 standard. The recrystallisation temperatures of recycled LLDPE and LDPE were 109 °C and 107 °C. The recycled materials were received in pellets form of ~2–2.5 mm size range. The MFI of V_LLDPE was 10 g/10 min, and the recrystallisation temperature was 108.85 °C. Food-grade CA and NaB were purchased from local suppliers in powder form. In addition, azodicarbonamide (ADC) blowing agent powder was supplied by Sigma-Aldrich, Victoria, Australia, and used as a non-biodegradable and potentially toxic agent to be compared with CA and NaB, which are more eco-friendly agents. Cryogenic pulverisation of the recycled LLDPE, LDPE, and citric acid were conducted using liquid N2 on an Omni Macro ES, while the NaB and V_LLDPE were obtained in finely powdered form from origin. Comparisons were made with a PU foam mattress obtained from A.H. Beard Pty Ltd., Derrimut, Victoria, Australia. The foams from the recycled and virgin polyolefins were fabricated using CA, NaB, and ADC using both low-shear pulverising and high-shear internal mixing with the methods detailed below.

a. Low-shear pulverising:

1. The pulverised recycled LDPE, recycled LLDPE, and V_LLDPE were dry blended in an Omni Macro ES for up to 15 min with 15% by weight of CBA (whether CA or NaB).
2. A total of 6 g of the dry blended and pulverised mix was placed in a specially designed mould (Figure 2a).
3. The entire assembly was placed in a Thermoline Scientific Oven at 200 °C for 15 min until foaming was achieved.
4. The sample was withdrawn from the oven, removed from the mould, and cooled to RT and used for further analysis (Figure 2b).

b. High-shear internal mixing

**Figure 3:**
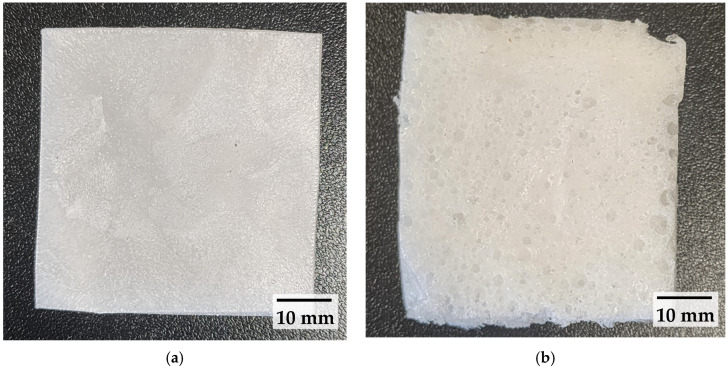
(**a**) Dough obtained after internal mixing of V_LLDPE with NaB. (**b**) Foamed polymer obtained from dough.

1. Recycled LDPE, recycled LLDPE, or V_LLDPE with 15% by weight of CBA (CA or NaB) were introduced into a Thermo Scientific internal mixer operated at 130 °C and 50 rpm for 5 min. However, ADC was added in 2.5, 5, and 10 wt.% to LDPE and was mixed for 7 min at 110 °C.
2. The dough obtained (Figure 3a) was then placed in the mould, as in Figure 2a, and steps a3-a4 were repeated until a foam was obtained, as in Figure 3b.

Thermal decomposition of the foaming agents was conducted on a TA Q 50 Thermogravimetric Analyzer (TGA) at a heating rate of 10 °C/min in air. Thermal properties of the fabricated foams were measured on a TA Differential Scanning Calorimeter (DSC). DSC testing began by heating the material to 200 °C with a heating rate of 10 °C/min followed by cooling down to 20 °C at the same rate. Peaks corresponding to the melting and recrystallisation temperature were noted. In addition, using the heat of fusion for a 100% crystalline PE as 293.1 J/g [22], the overall crystallinity was calculated using the following equation (Equation (4)), where ∆Hm is the fusion enthalpy (J/g) calculated for each composition using the obtained graphs, and $∆H_{m}^{0}$ is the fusion enthalpy for 100% crystalline PE (293.1 J/g).

(4) $$X_{c}=\frac{∆H_{m}}{∆H_{m}^{0}}\times 100\%$$

Microstructural analysis of the foams was conducted on an Olympus BX61 optical microscope. This was used to both image the microstructure of the fabricated foams and estimate the porosity in the form of pore sizes, as well as to span and to be compared with commercial PE and PU foams.

Standard shapes were cut from the foam specimens, and their mass and volume were estimated to calculate the density of the fabricated foams. Using the density values, the %porosity ($V_{P}$) as measured in Prasad et al. [23] was as shown in Equation (5), where $\rho_{f}$ and $\rho_{u}$ are densities of the material post and pre foaming, respectively. $V_{P}$ was then used to determine the efficacy of the foaming procedure.

(5) $$V_{P}=\left(1-\frac{\rho_{f}}{\rho_{u}}\right)\times 100\%$$

Mechanical properties of the prepared foams using NaB and ADC were compared in terms of compression set under constant deflection and compressive strength according to the ASTM D3575 standard [24]. The test specimens were compressed to their 50% of their original thickness ($t_{o})$ and remained deflected for 22 h. The final thickness ($t_{f})$ was measured after 24 h of recovery. All tests were performed using a Zwick Z010 (Germany) universal testing machine when a 10 kN load cell was mounted. The compression set values ($C_{d})$, as a percentage of the original thickness were then calculated for each sample using Equation (6).

(6) $$C_{d}=\frac{\left(t_{o}-t_{f}\right)}{t_{o}}\times 100\%$$

## 3. Results and Discussion

The results of the experimental studies on the base materials and foam samples morphology; the thermal properties and mechanical are presented in this section.

### 3.1. Morphology

**Figure 4:**
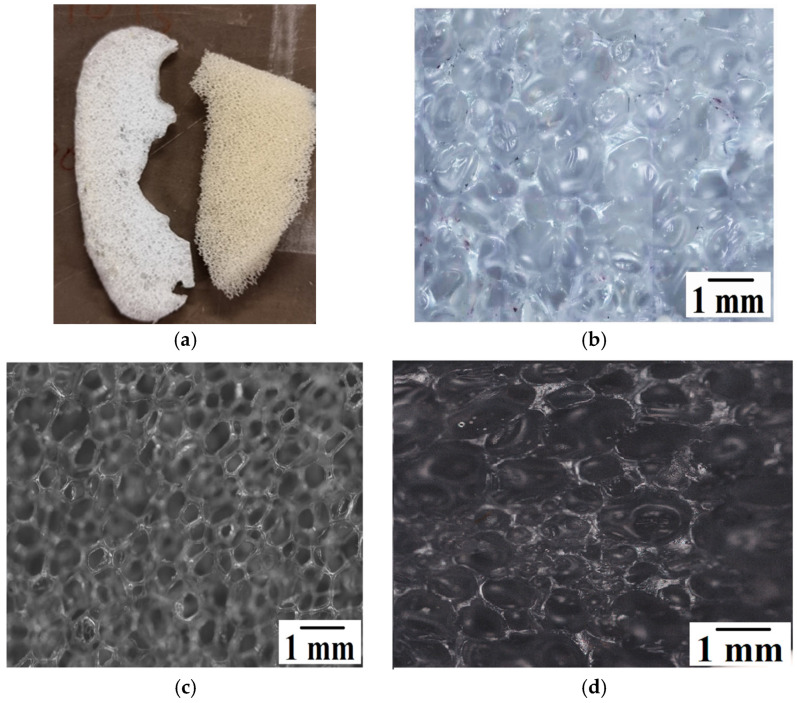
(**a**) Comparison of V_LLDPE CA pulverised foam with commercial PU foam. Optical microscopy images of (**b**) V_LLDPE CA pulverised foam; (**c**) commercial PU foam; (**d**) V_LLDPE CA batch mixed foam.

A side-by-side comparison of the foam fabricated using CA and V_LLDPE with a commercial PU foam is presented in Figure 4a. The corresponding comparison under optical microscopy is shown in Figure 4b–d. A few observations that can be made from Figure 4 are as follows:

1. The pores observed for the commercially available foamed PU sample, which were used in mattresses, were of an open cell variety, while the ones for the V_LLDPE foam were of the closed cell variety.
2. The shapes of the pores in the commercial foam were polygonal in nature, while the pulverised V_LLDPE CA and batch mixed V_LLDPE CA foam exhibited mostly spherical pores.

**Figure 5:**
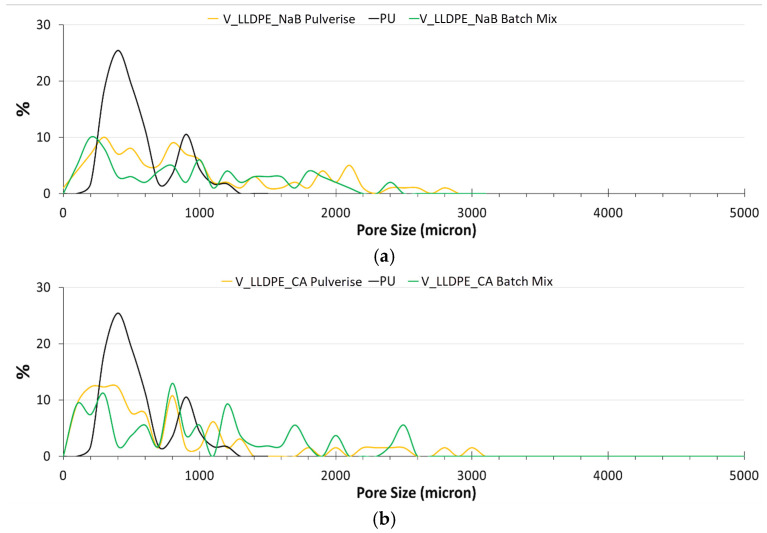
Comparison of the pore size distribution of pulverised and batch-mixed foams of V_LLDPE produced with different chemical foaming agents: (**a**) NaB; (**b**) CA.

**Figure 6:**
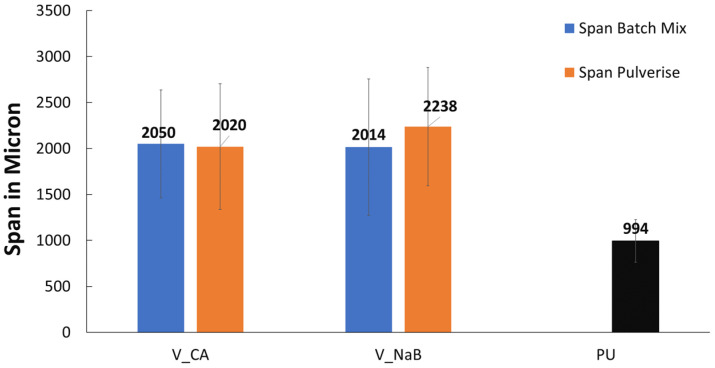
Span of pore sizes observed in V_LLDPE foams made from CA and NaB under pulverisation and batch mixing conditions benchmarked with PU mattress foam.

An overall comparison of the virgin polyolefin foams with commercial PU mattress foam in terms of pore size distribution is shown in Figure 5. There was a pronounced multi-modal size distribution of the pores in both the PU and the V_LLDPE foams but while nearly 95% of the pores in the PU foam were found to be in the less than 1000 μm diameter region, the V_LLDPE foams showed pores up to 3000 μm in size. This size distribution of the pores was broader for the V_LLDPE foams, irrespective of the use CA or NaB as the foaming agent or even the use of internal mixing or under pulverisation conditions (Figure 6).

It has to be noted that as a consequence of the broad size distribution, the % of pores less than 100 μm was also much higher for the V_LLDPE foams than the PU foams, and therefore, with more stringent constriction of flow, the pore coalescence can potentially be controlled, and the size distribution of the pores can be reduced for the CA and NaB V_LLDPE foams. However, it is also worth noting that the inherent stiffness of the V_LLDPE foam was higher than the PU foam. Interestingly no major effect was observed for the span in pore sizes, irrespective of the foaming agent used or the method of application of shear. This could have been because the V_LLDPE used was a rotomolding grade with a very low zero-shear viscosity, meaning mixing with another powdered material of a similar size distribution could lead to effective dispersion, even in the absence of high shear as long as a temperature stimulus was provided, as was seen in Prasad et al.’s [23,25,26] demonstration of wood composite fabrication with retainable mechanical properties using V_LLDPE, pine, and oak flour through comparatively low shear processing techniques such as compression and rotational moulding.

**Figure 7:**
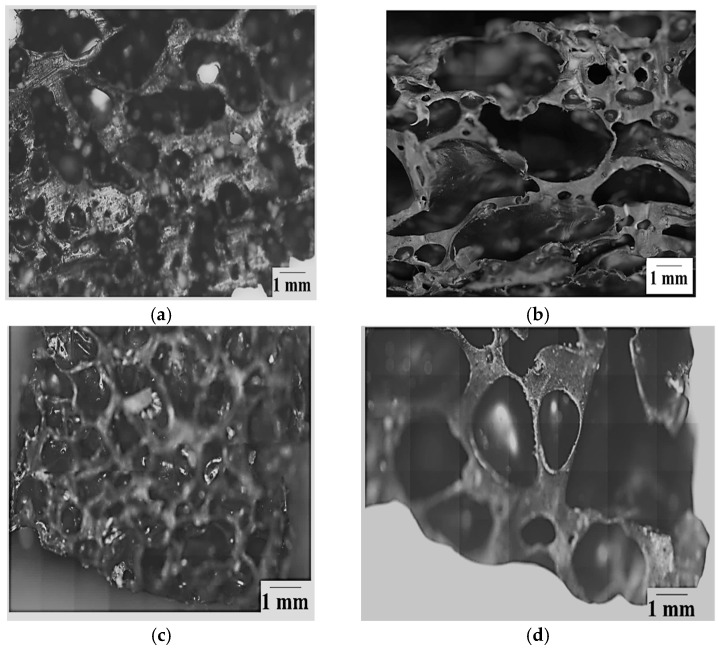
Batch mixed CA foam: (**a**) LDPE; (**b**) LLDPE, pulverised NaB foam; (**c**) LDPE; (**d**) LLDPE.

**Figure 8:**
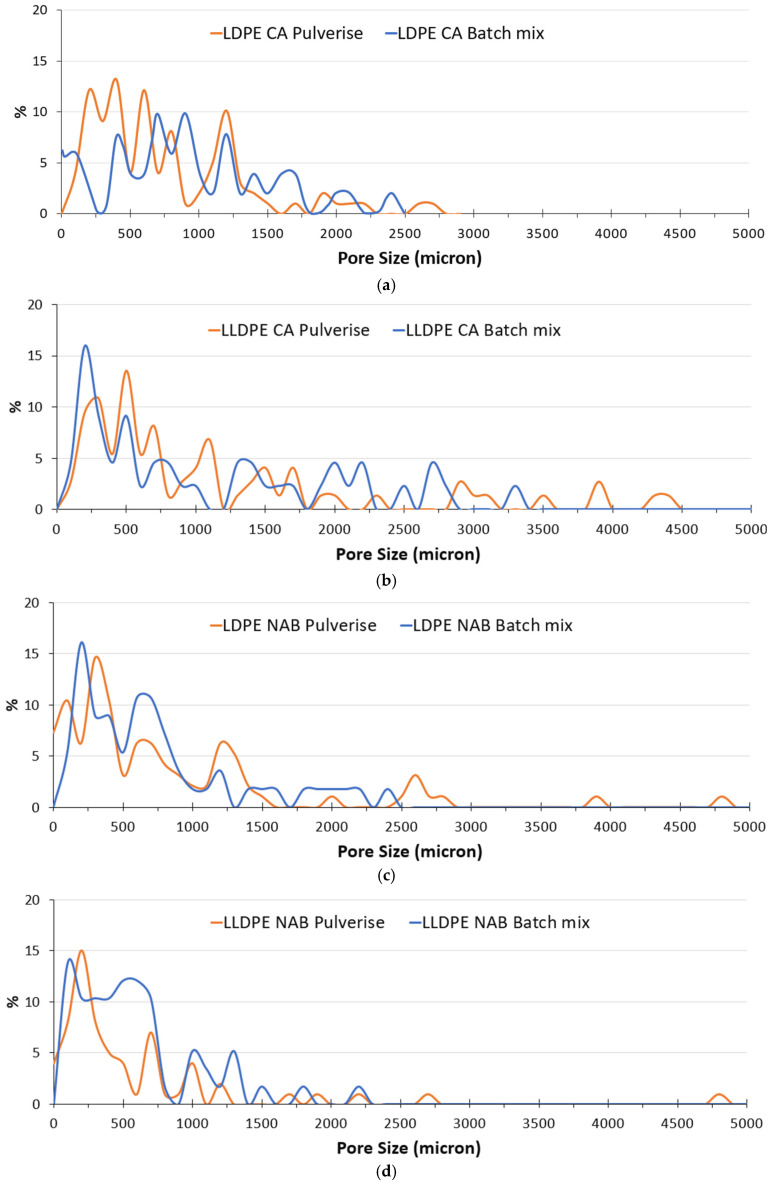
Pore size distributions from the microstructure of recycled polyolefin foams: (**a**) LDPE with CA foams; (**b**) LLDPE with CA foams; (**c**) LDPE with NaB foams; (**d**) LLDPE with NaB foams.

**Figure 9:**
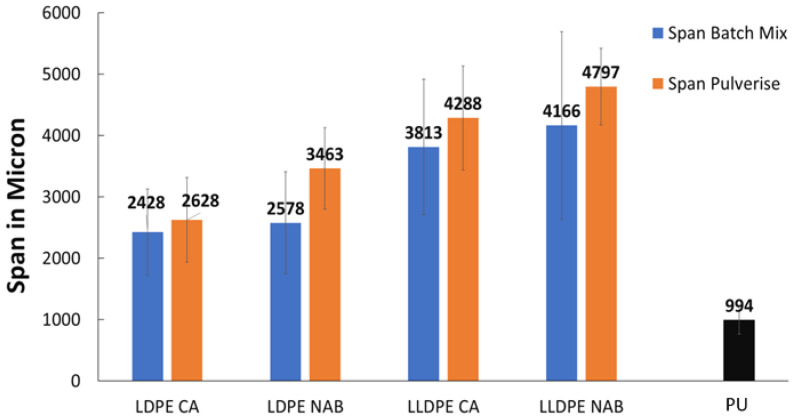
Span of pore sizes observed in recycled LLDPE, and recycled LDPE foams made from CA and NaB under pulverisation and batch mixing conditions benchmarked with PU mattress foam.

Through the analysis of foams fabricated from recycled polyolefins (Figure 7), the influence of the process and type of foaming agent on the foaming phenomenon could be uncovered in greater detail. It could be seen that the span of pore sizes reduced when the higher shear batch mixing was used as opposed to pulverisation mixing (Figure 8 and Figure 9). In addition, the use of CA generally led to lower pore sizes and narrower size distributions in terms of span than the use of NaB (Figure 9). This could be explained based on the mechanism of foaming agent decomposition. From Equations (1) and (2), it can be seen that the decomposition reaction for NaB was much simpler than the complex CA mechanism. In addition, the CA mechanism also proceeded through an additional step of phase change, i.e., the CA had to melt before the decomposition reaction could occur. Therefore, a greater degree of control can be exerted on the foam fabrication using CA as the foaming agent than the one-step, no phase change NaB decomposition reaction.

Above being said, it was clear from both the microscopy images and the pore size distribution data that the recycled LLDPE-based foam showed much more coalescence than the recycled LDPE-based foam, irrespective of the type of foaming agent or the use of pulverisation or batch mixing methods. It can be speculated that there is a range of reasons behind this variation in behaviour between the two recycled polyolefins.

The main driving force behind the ability of materials such as CA and NaB to create foamed microstructures is the nature of the decomposition reactions, as explained earlier, but it is also true that the presence of additives and unaccounted for impurities in the polymers can have unpredictable effects on these reactions. This may be why there is such behaviour seen for the recycled LLDPE, while the V_LLDPE on the other hand did not show either pronounced pore coalescence or strong variation depending on the type of foaming agent used. In addition, the gradient of concentration of such impurities and secondary phases could be much higher within the LLDPE as compared to the recycled LDPE, and this could also contribute towards the tendency of the recycled LDPE that seems to show a more controlled size distribution and less pore coalescence as compared to the recycled LLDPE.

Thirdly, as compared to the V_LLDPE and recycled LDPE, the recycled LLDPE was biphasic in nature, as seen from the DSC melting data shown in Figure 11 and Figure 12. The presence of a PP phase could act as a controlling factor towards nucleation and prevent pore coalescence, but there is a precedent set in the study [27] where at higher concentrations of PE (75% by weight and above), there may be a partial miscibility of the two polymers, viz., PE and PP manifesting itself by a shouldered crystallisation peak, as seen in Figure 11c and Figure 12b. This co-miscibility could then equalise the relative interactions between the PE and PP phases with the foaming agent, encouraging pore coalescence.

### 3.2. Thermal Properties

**Figure 10:**
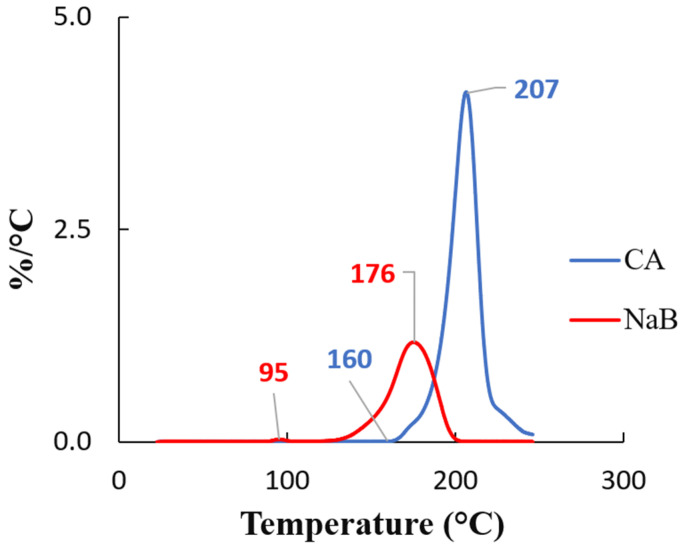
DTA graphs of the foaming agents NaB and CA showing the peak decomposition temperatures.

The decomposition reactions shown in Equations (1) and (2) can also be visualised through differential thermal analysis (DTA) of the foaming agents, as seen in Figure 10. Both materials had overlapping reactivity around 200 °C, with the reaction of the NaB attaining completion faster, whereas the CA was still on an upward trend until its full decomposition. Noting this, a decision was made to cap the foaming reaction at 200 °C, owing to the lowered thermal stability of the V_LLDPE and recycled LDPE and recycled LLDPE beyond 200 °C. The TGA analysis of the recycled polymers are presented in Figure S1 (Supplementary Materials).

**Figure 11:**
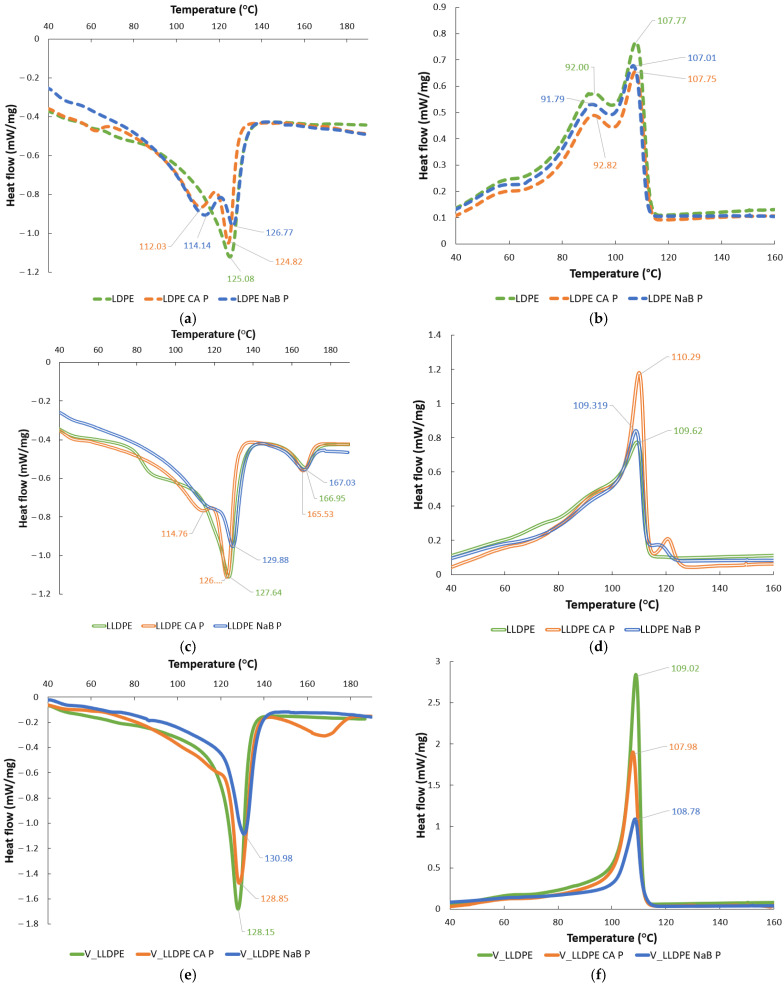
DSC curves of pulverised foam materials: (**a**) melting curve of LDPE foams; (**b**) recrystallisation curves of LDPE foams; (**c**) melting curve of LLDPE foams; (**d**) recrystallisation curves of LDPE foams; (**e**) melting curve of V_LLDPE foams; (**f**) recrystallisation curves of V_LLDPE foams.

**Figure 12:**
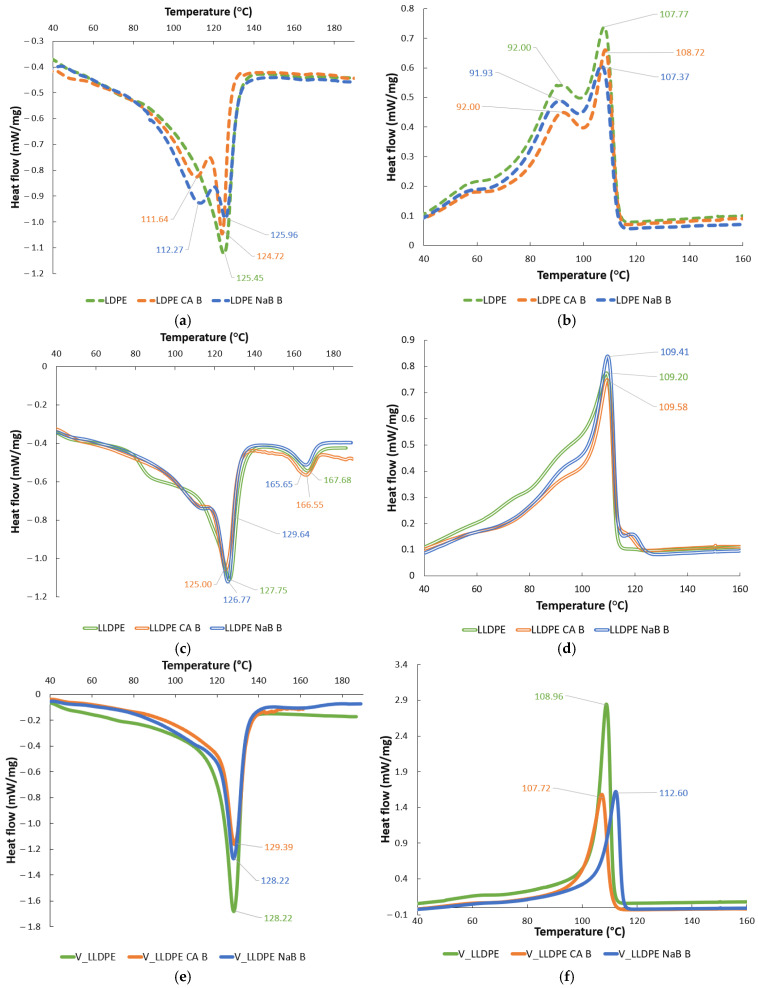
DSC curves of batch mixed foam materials: (**a**) melting curve of LDPE foams; (**b**) recrystallisation curves of LDPE foams; (**c**) melting curve of LLDPE foams; (**d**) recrystallisation curves of LDPE foams; (**e**) melting curve of V_LLDPE foams; (**f**) recrystallisation curves of V_LLDPE foams.

**Figure 13:**
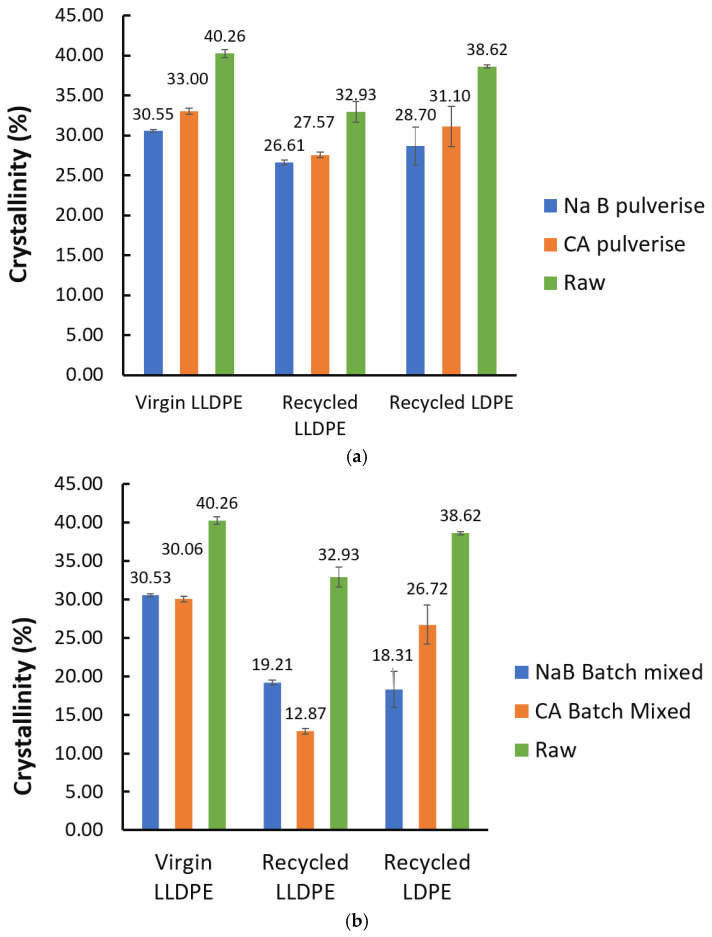
Degree of crystallinity values of all fabricated foams: (**a**) via pulverisation; (**b**) via batch mixing.

A more thorough thermal analysis could help ascertain the structural effects of foaming on the polymer materials, and to this end, DSC analysis was conducted, as shown in Figure 11 and Figure 12, for the pulverised and batch mixed foams, respectively. A review of the literature indicates that the work conducted on the thermal characterisation of recycled-polyolefin-based foams is relatively low, but some general observations can be made:

1. It can be clearly seen that melting peak became broader and less sharp, which indicated that a range of crystal sizes formed during the foaming process for all the polymers [28].
2. The recrystallisation peaks showed changes in shape and sharpness and also exhibited new peaks indicative once again of the different crystal sizes and mechanisms initiated during and after the foaming process.
3. This also affected the overall polymer crystallinity, as seen in Figure 13. It can be seen that there was a drop in crystallinity throughout after foaming, irrespective of the mixing method or foaming agent used. This was partly due to the widening of crystallite sizes and forms, as reported in [28]. In addition, the presence of the solid foaming agent particles can limit crystallite’s growth in spite of higher crystallite seed density. The quantity of reported work in this specific topic, i.e., the competition between cell nucleation in foaming and crystal nucleation for semi-crystalline polymers like LLDPE and LDPE is limited, and hence this is speculative at the moment [29]. However, it can be seen from our results in Figure 13 that the NaB foaming agent that stayed solid throughout the foaming process resulted in lowered crystallinity than CA, which underwent a phase change to liquid prior to decomposition and foaming.

**Figure 14:**
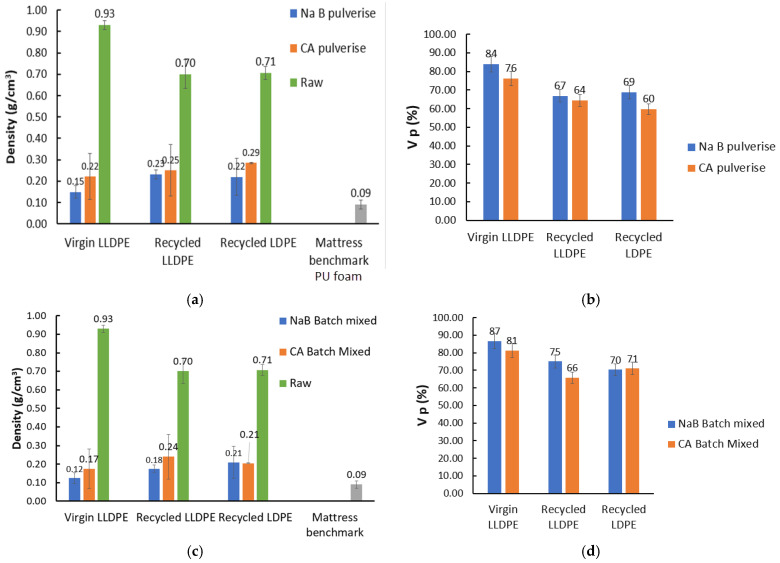
Densities and V_P_ of manufactured foams vs. raw materials with PU mattress benchmark: (**a**,**b**) via pulverisation; (**c**,**d**) via batch mixing.

One of the most critical properties of a foam material is its density, as established in a wide range of studies on the topic [17,18,19,20,21,30,31,32]. In these studies, the average pore diameters in PU foams were from 75 to 600 μm [30,31,32], and the benchmark foam used in this work had an average pore diameter of about 200–300 μm, as seen in Figure 5. While the foams produced in this work also showed similar pore sizes, the span was much wider, as discussed, and also another point of variation is the density. As reported by [30,31,32], the densities of PU foam mattresses range from 0.035 to 0.100 g/cm^3^, with our benchmark PU foam showing a density closer to the higher end of that spectrum at 0.09 g/cm^3^. The density values seen for recycled polyolefin foams tended to fall in the 0.13 to 0.91 g/cm^3^ range [17,18,19,20,21]. The density of the foams produced in this work were in that range as well. The foams produced from the V_LLDPE seemed to show a lower density than the recycled polyolefins. The reasons for these have not been explored as much in the literature, so, at this point of time, we can speculate that this is owing to the unquantified additives and impurities found in recycled plastics competing with and/or reacting with the foaming agents and reducing their efficiency. It can be seen that the NaB foams showed lowered densities (Figure 14a,c) and, as a consequence, higher V_P_ compared to the CA foams (Figure 14b,d), and this can be attributed to the larger pores seen with the NaB foams. However, this can also be because the NaB decomposition mechanism is a simple one-step process and driven through an ionic mechanism [33]. The mechanism of CA decomposition is much more complex and has many radical pathways [34]. These generated radicals may be consumed by the unaccounted-for antioxidant and heat stabiliser materials present in comingled/recycled polyolefin blends, leading to reduced efficiency of decomposition.

### 3.3. Mechanical Properties

**Figure 15:**
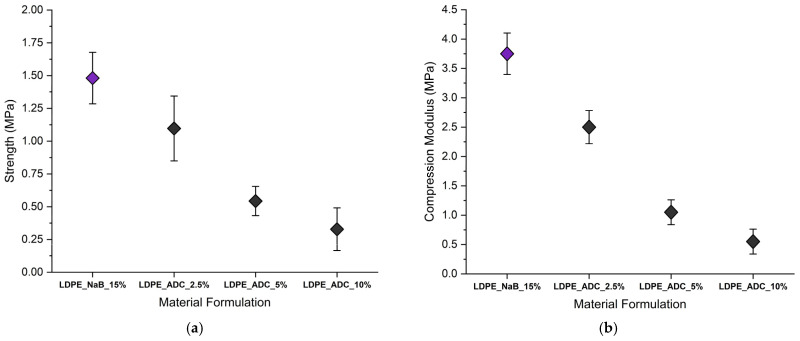
(**a**) The required strength for compressing the foams to 50% of the original thickness for different compositions. (**b**) Compression modulus of the tested foam samples.

The compression set under constant deflection and compression deflection (compressive strength) of the tested foams was measured for four sets of foams, namely, LDPE_NaB_15%, LDPE_ADC_2.5%, LDPE_ADC_5%, and LDPE_ADC_10%. The results of compressive strength tests are presented in Figure 15a,b. The horizontal axis shows the foam material formulation, while the vertical axis shows the compressive strength at 50% compression of the original thickness. The samples prepared using LDPE and NaB had the highest compressive strength with a mean value of 1.48 MPa, while the samples prepared using ADC foaming agent had lower compressive strength. The results also suggest that increasing the ADC content in LDPE reduced the compressive strength of the prepared foam samples.

**Figure 16:**
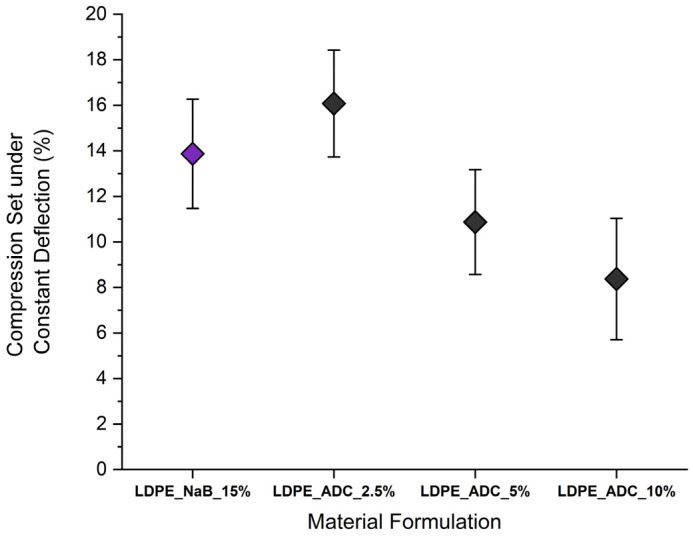
Compression set under deflection of 50% for LDPE foams with different compositions.

The results of the compression set under constant deflection for the tested foams are presented in Figure 16. The highest compression set was measured for LDPE_ADC_2.5% at 16.1%, while the increase in ADC content enhanced the foam recovery as compression set values decreased to 10.8% and 8.4%, respectively, for 5% and 10% ADC content. Although LDPE_NaB_15% samples recorded the highest rigidity according to Figure 16, they had higher resilience compared to LDPE_ADC_2.5% samples, with the mean compression set value of 13.9%.

## 4. Conclusions

Low-cost foaming agents CA and NaB were used under conditions of pulverisation of high-shear internal mixing to fabricate foams from virgin and recycled polyethylenes and benchmarked against a commercially available PU foam. While pore size and distribution are critical parameters for a foam’s performance, it was found that the benchmark PU foams also showed pore shapes that were more polygonal than the mostly spherical pores produced using CA and NaB. The mechanism of decomposition also had an influence on the foam characteristics. The more complex radical decomposition mechanism of CA, which required the phase change prior to decomposition, led to foams with narrower pore spans and narrower size distributions, but at the cost of slightly higher densities and hindered foaming efficiency, owing to the unaccounted-for additives (antioxidants, heat stabilisers, etc.) in the recycled polyolefins. The simple, ionic mechanism of decomposition of the NaB lent itself to larger pores, lowered density, and less control on the pore size distribution. Overall, the span of pore sizes reduced when the higher shear batch mixing was used as opposed to pulverisation mixing. This study demonstrated important key steps towards valorisation of waste polyolefin compounds through chemical foaming for potential use in circular packaging applications.

Future work will focus on optimising processing parameters (temperature, mixing speed, and time) to improve foam production consistency. Alternative blending techniques and additives can enhance mechanical performance of the foam, while minimising impurities in recycled materials will improve process reproducibility. Additionally, further studies on cyclic compression performance and other mechanical durability studies will better inform the foams’ suitability for practical applications.

## Data Availability

The data presented in this study are available upon reasonable request from the corresponding authors.

## Supplementary Materials

The following supporting information can be downloaded at https://www.mdpi.com/article/10.3390/polym17091270/s1, Figure S1: TGA analysis of recycled LLDPE and LDPE: (a) changes of weight vs. temperature and (b) differential thermal analysis (DTA) curve.
