# Supplementary material for: An Analysis of Foams Produced from Recycled Polyolefins and Low-Cost Foaming Agents: Benchmarking Using Pore Size, Distribution, Shear Effects, and Thermal Properties
Source: Polymers (Basel). 2025 May 6;17(9):1270. doi: 10.3390/polym17091270 (PMC12074132; doi:10.3390/polym17091270)
Supplement: Supplementary file 1 [file polymers-17-01270-s001.zip › polymers-3530296-supplementary.pdf]

Article

An analysis of foams produced from recycled polyolefins and low-cost foaming agents: Benchmarking using pore size, distribution, shear effects and thermal properties

Krishnamurthy Prasad [1], Fareed Tamaddoni Jahromi [1], Shammi Sultana Nisha [1], John Stehle [2], Emad Gad [1], Mostafa Nikzad [1,*]

[1] School of Engineering, Swinburne University of Technology, Hawthorn, VIC, 3122, Australia; krishnamurthyprasad@swin.edu.au (K.P.); ftamaddonijahromi@swin.edu.au (F.T.J.); snisha@swin.edu.au (S.S.N.); egad@swin.edu.au (E.G.)
[2] Robovoid Pty Ltd, Australia; johnstehle@robovoid.com (J.S.)
* Correspondence: mnikzad@swin.edu.au (M.N.)

Supplementary Material S1

Thermogravimetry analysis of the recycled polymers

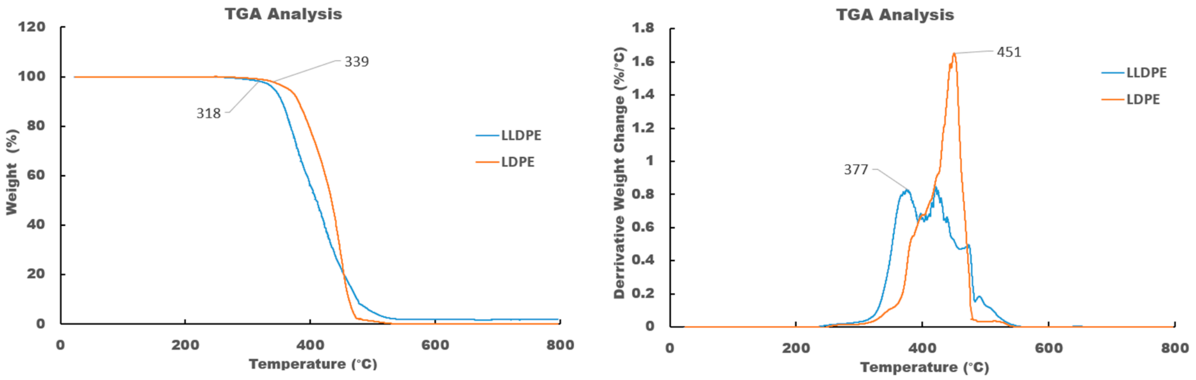

(a) (b)

Figure S 1 TGA analysis of recycled LLDPE and LDPE, a) changes of weight vs temperature and, b) Differential Thermal Analysis (DTA) curve

 https://doi.org/10.3390/xxxxx
